# The Constructive Role of Teacher Enthusiasm and Clarity in Reducing Chinese EFL Students’ Boredom

**DOI:** 10.3389/fpsyg.2022.874348

**Published:** 2022-04-07

**Authors:** Yang Song

**Affiliations:** School of Foreign Studies, Henan Agricultural University, Zhengzhou, China

**Keywords:** positive psychology, teacher enthusiasm, teacher clarity, interpersonal communication skills, students’ boredom, EFL

## Abstract

With the rise of positive psychology, the role of teachers’ emotions and interpersonal communication skills has been recently highlighted in the literature. However, the preventive role of teacher enthusiasm and clarity in reducing EFL students’ boredom has not caught sufficient attention among L2 scholars. Against this gap, this article, first, presented the definitions, dimensions, and conceptualizations of teacher enthusiasm, clarity, and students’ boredom. Next, theoretical and empirical backgrounds were provided to support the claim that enthusiasm and clarity of EFL teachers can stop students’ classroom boredom. Additionally, the study presented the implications and future directions of this line of research for different people such as EFL teachers, teacher trainers, and L2 scholars. The ideas can improve their awareness of teachers’ positive emotions, interpersonal skills, and their roles in L2 education.

## Introduction

Despite the fact that there have been made various improvements and progressions in having a better educational system/curriculum concerning second/foreign language education in China, there are still many challenges and setbacks that need to be considered and tackled wisely (Peng, 2021). The rapid growth of learning English has led to the establishment of numerous academic centers trying to increase the proficiency of Chinese EFL students (Wang, 2017). However, there appeared the problem of teaching with a low quality and efficiency in China’s L2 education demanding a swift shift from quantity extension to quality improvement. Aiming to solve this issue, significant attempts have been made by the associated stakeholders in China to monitor the quality of instruction focusing on instructional facilities and teachers’ methodology and techniques to teach English. Moreover, with the emergence and popularity of positive psychology (PP), the role of emotions in L2 education has captured a surge of interest among Chinese EFL teachers and researchers (Wang, 2017; Li, 2020). The focus was converted from negative stressors to positive emotions in education and their power to generate several optimum outcomes (MacIntyre et al., 2019).

Such outcomes are achievable in a classroom where the teacher takes learners’ emotions and inner drives into consideration and forms a positive interpersonal interaction with them (Wang et al., 2021; Xie and Derakhshan, 2021). Yet, simply caring for students’ emotions is not ample for academic success and there must be a representation of that care in teachers’ instructional behaviors and practices as well. One of the venues for this concerns teachers’ own positive emotions like enthusiasm in teaching that is a fundamental feature of good teachers and a cause of student learning (Kim and Schallert, 2014; Lazarides et al., 2019). It is a simultaneous demonstration of positive affective engagement and the behavioral manifestation of that engagement in teaching (Keller et al., 2018). In simple terms, the concept of teacher enthusiasm concerns the emotional experience of joy and pleasure during instruction and its behavioral display or transmission (Frenzel et al., 2009). Research reveals that this constructive teacher-related factor increases students’ motivation, engagement, academic achievement, and attention (Zhang, 2013; Peng, 2021).

Another related expressive sign of teacher enthusiasm is clarity of interaction that is transmittable to students (Frenzel et al., 2009). Teacher clarity is one of the most important interpersonal communication skills that pertain to teachers’ utilization of different strategies and approaches to ensure that their students have learned the content of the course (Bolkan, 2017). Clarity is demonstrated in teachers’ classroom organization, descriptions, examples, techniques, and assessment (Zheng, 2021). In L2 contexts that are now significantly interaction-based, teachers’ interpersonal communications skills like clarity have a crucial role in determining and improving learners’ cognitive learning, success, engagement, interest, agency, and motivation (Titsworth et al., 2015; Bolkan, 2017; Xie and Derakhshan, 2021; Zheng, 2021). Aside from these benefits, in the classroom, as the cornerstone of gaining academic outcomes, teacher enthusiasm and clarity are two prominent positive emotions that can reduce the impact of negative emotions like boredom as well. Boredom is an aversive emotion that is a blend of other negative stressors such as disengagement, dissatisfaction, inattentiveness, amotivation, low energy, and inaccurate time perception (Pawlak et al., 2020, 2021). It has deleterious impacts on L2 education while it lacks an overt indication to be identified and dealt with Derakhshan et al. (2021a,b) and Zawodniak et al. (2021). The concept has recently gained attention in EFL contexts, yet it requires more empirical studies on its predictors, sources, solutions, and correlated variables. In tune with these, the current mini-review research aimed to investigate the effect of Chinese EFL teachers’ enthusiasm and clarity on students’ level of boredom in L2 classrooms.

## Background

### Teacher Enthusiasm

The concept of teacher enthusiasm was first introduced in 1970s when it was considered as an instructional behavior including non-verbal communication behaviors such as smile, eye contact, gesture, facial expressions, and the like. This led to a uni-dimensional approach to the definition of enthusiasm making it equated with teacher enjoyment (Kunter et al., 2011). This conceptualization regards teacher enthusiasm as quality of effective teaching that affects learners’ academic performance by indicating more energy and interest in the subject, the contents, and their presentation in a dynamic, motivating way. In this perspective, enthusiasm pertains to teachers’ expressiveness and capability to convey the significance and inherent value of the materials to their pupils (Kim and Schallert, 2014; Wang and Guan, 2020).

The other perspective that is bi-dimensional perceives teacher enthusiasm as an emotional-behavioral characteristic involving individual disposition and emotion (Kunter et al., 2011). It is clarified as the affective experiences of enjoyment, excitement, and pleasure together with their expressive behaviors that are actually occurring and manifesting such experiences in the classroom (Kunter et al., 2011). This recurring emotion can be related to the subject matter and the teaching activity. Moreover, the construct of teacher enthusiasm like other positive emotions is claimed to be contagious in that it can be easily transmitted to students whose motivation, vitality, attention, engagement, and interest are correspondingly improved by this factor (Zhang, 2013; Lazarides et al., 2019; Peng, 2021).

### The Constituents of Teacher Enthusiasm

Teacher enthusiasm in the class involves various behaviors and practices. Hence, it includes different components as evidenced in the literature. For instance, eight constituents for the construct of teacher enthusiasm include: (1) varying speed and tone of voice, (2) maintaining eye contact, (3) using demonstrative gestures, (4) body movement, (5) showing a lively facial expression, (6) using illustrative words, (7) showing willingness to accept students’ ideas and feelings, and finally (8) maintaining vitality. In a similar vein, the following nine elements shape a teacher’s enthusiasm (Table 1).

**Table 1:** The components of teacher enthusiasm.

#	Component
1	Speaking in a dramatic expressive way
2	Variation in pitch and volume
3	Vocal inflection
4	Smiling or laughing while teaching
5	Moving about while lecturing
6	Gesturing with hands or arms
7	Exhibiting facial gestures or expressions
8	Eye contact
9	Humor

Both these studies considering the constituents of teacher enthusiasm have been conducted decades ago meaning that recent studies on the construct have turned a blind eye on the new possible components in EFL contexts and across cultures. This can be a direction for future research aiming to add to or modify the existing components of teacher enthusiasm.

### Teacher Clarity

The concept of teacher clarity is a relational behavior belonging to interpersonal communication skills (Comadena et al., 2007; Xie and Derakhshan, 2021). As pinpointed by Bolkan (2017) and Comadena et al. (2007), the construct of teacher clarity pertains to both verbal and non-verbal cues, strategies, and approaches that teachers utilize to ensure that their pupils have mastered the content and processes of the course. Hence, having verbal and non-verbal clarity is essential for EFL teachers to inspire meaning in the minds of the students regarding the course, its content, processes, and purposes (Bolkan, 2016; Zheng, 2021). There is enough research evidence that teacher clarity causes various positive academic outcomes such as students’ increased motivation, classroom engagement, participation, attendance, critical thinking, empowerment, and immediacy (Finn and Schrodt, 2012; Titsworth et al., 2015; Bolkan, 2017; Zheng, 2021). Another probable benefit of teacher clarity in EFL contexts relates to the reduction and removal of negative variables like boredom as clarity generates course comprehension, positive rapport, credibility, and academic interest. As a result, students rarely get entangled in the tedium and tiredness of the class in case they have a clear teacher who strives to engage students in the course process and content (Zheng, 2021).

### The Rhetorical/Relational Goal Theory

As one of the instructional communication theories, the Rhetorical/Relational Goal Theory (RRGT) that was introduced by Mottet et al. (2006) aims to reveal the nature and operational process of instructional communication. Moreover, the theory posits that classroom teachers and learners have rhetorical and relational goals to which they wish to reach (Mottet et al., 2006). Consequently, each classroom draws on the needs/goals of both students and their teacher who have relational needs (e.g., to be admitted) and rhetorical needs (e.g., to complete a task and to get a high grade). This is the duty of teachers to simultaneously manage these needs *via* their behavioral choices in the classroom (Frymier, 2007). It is believed that teachers’ rhetorical and relational behaviors serve diverse purposes. For example, they may use “clarity” as a rhetorical instructional communication behavior to improve the quality of teaching and influence students’ beliefs, behaviors, and practices in the classroom through determining their intended instructional messages (Beebe and Mottet, 2009). Teachers may also use rhetorical and relational behaviors to establish a positive rapport with their students in the classroom (Myers, 2008).

In sum, it can be argued that in any educational setting, teachers need to employ a combination of rhetorical and relational behaviors to produce positive outcomes (Myers et al., 2018). Those behaviors can be influenced by other factors like teaching context, teachers’ personality, credibility, clarity, immediacy, homophily, and learners’ perceptions (Beebe and Mottet, 2009; Zheng, 2021). Yet another teacher-related factor that can affect classroom rhetorical and relational behaviors is teacher enthusiasm and its role in reducing students’ negative emotions (e.g., boredom), which has been limitedly explored in L2 contexts.

### The Concept of Boredom: Definitions and Dimensions

The concept of boredom is very complicated and hard to be defined by a single characteristic. It is a combination of feelings such as disengagement, dissatisfaction, lack of attention, amotivation, demotivation, lack of energy, and wrong time perception (Pawlak et al., 2020). Consequently, offering a one-size-fits-all definition for it has long been impossible. This might be due to its unobservable and multi-layered nature and similarity to other terms such as sluggishness, inactivity, tedium, and indifference (Daniels et al., 2015; Weinerman and Kenner, 2016). However, most researchers in this domain certify that boredom is a damaging, negative, silent, fleeting, deactivating, and disappointing emotion affecting learners’ learning process (Li et al., 2021). It ruins the pleasure of teachers’ and learners’ practice and generates disengagement and frustration that finally end in poor academic performance.

With regard to its dimensions, boredom has been claimed to include three dimensions of valence, activation/arousal, and objective focus. Valence refers to the amount of pleasantness/unpleasantness of a feeling, while activation concerns the physical/cognitive activation/deactivation of an emotion. Finally, objective focus concerns if the feeling is activity-oriented or outcome-oriented (Pekrun, 2006).

### Sources and Coping Strategies of Boredom

Boredom emerges and takes root from various factors and sources such as those related to students, teachers, materials, and equipment (Li, 2021). As for student-related factors, low language proficiency, motivation, interest, fatigue, and a negative appraisal are the key sources of boredom. Concerning teacher-related causes, poor teaching abilities, untimed feedback, boring speech/presentation, and poor classroom rapport are the sources of boredom. Furthermore, monotonous and unchallenging classroom tasks and materials are task-related sources of boredom. Finally, inadequate amenities and tools in the class (e.g., digital tools, audio-visual tools, whiteboards, and markers) may generate disengagement and boredom among teachers and students (Daniels et al., 2015; Derakhshan et al., 2021a,b; Li, 2021).

Despite these challenges and causes, students and teachers can employ many strategies such as analyzing issues from a different outlook, utilizing new resources, resorting to activities that require physical activity in the class (e.g., standing up, walking, and using music; Kruk and Zawodniak, 2018). As boredom is new in L2 education, few studies have yet been conducted on this critical emotion being limited to unraveling its causes and solutions. However, more studies are needed in this area to see if teacher-related variables like enthusiasm and clarity can prevent students’ boredom in English classes or not.

## Concluding Remarks

In this mini-review study, an attempt was made to present the theoretical and empirical underpinnings of two pivotal teacher-related factors (enthusiasm and clarity) in their influential role in stopping and minimizing students’ boredom in EFL classrooms. It was contended that teachers’ experience, the manifestation of enthusiasm in the classroom, and suitable interpersonal communication skills (e.g., clarity) facilitate the ground for many positive academic outcomes and diminish negative stressors like boredom in L2 education. This is to argue that EFL teachers who have experienced joy and excitement in the class and express such feelings in their actual pedagogy are willing to be clear to their students and establish a positive classroom atmosphere by which success steps in. Hence, it can be stated that teacher-psychology variables and interpersonal communication skills are valuable coping strategies for reducing and removing boredom in L2 education. Based on these, this article can be beneficial for EFL teachers, teacher trainers, and researchers. EFL teachers can realize the importance of their enthusiasm and clarity in generating students’ success and removal of negative emotions in the class. More specifically, they can utilize sympathetic and engaging instructional practices that maintain students’ attention and impede boredom and subsequently enhance students’ motivation (Wang and Derakhshan, 2021). This sense of excitement also creates professional wellbeing in teachers as enthusiastic instructors are more prone to be fulfilled in their life and occupation (Buric and Moe, 2020). Teacher trainers can use this study to offer training courses and workshops to educate EFL instructors on developing their positive emotions and ways to be clear in the class to reduce negativity and boredom. Likewise, they can provide novice and experienced EFL teachers with various classroom techniques and strategies to add excitement, joy, enthusiasm, and clarity to the process of teaching and learning that end in full participation and engagement. Finally, SLA researchers can use the ideas of this review and conduct complementary studies on the role of teacher emotions in reducing other negative stressors of L2 education. To do so, they can use longitudinal, qualitative, and mixed-methods research designs or gather data from various cultural groups.

## Author Contributions

The author confirms being the sole contributor of this work and has approved it for publication.

## Funding

This study was supported by Humanities and Social Science Project administered by the Education Department of Henan Province in 2020 Research on Applied Translation Teaching and Talent Cultivation under the Background of the Belt and Road Initiative (no. 2020-ZZJH-193).

## Conflict of Interest

The author declares that the research was conducted in the absence of any commercial or financial relationships that could be construed as a potential conflict of interest.

## Publisher’s Note

All claims expressed in this article are solely those of the authors and do not necessarily represent those of their affiliated organizations, or those of the publisher, the editors and the reviewers. Any product that may be evaluated in this article, or claim that may be made by its manufacturer, is not guaranteed or endorsed by the publisher.
